# Exploring the longer-term impact of the COVID-19 pandemic on physical and mental health of people with inflammatory rheumatic diseases: a cross-sectional survey

**DOI:** 10.1007/s10067-023-06565-0

**Published:** 2023-03-07

**Authors:** Samantha Hider, Sara Muller, Lauren Gray, Fay Manning, Mike Brooks, Dominic Heining, Ajit Menon, Jonathan Packham, Edward Roddy, Sarah Ryan, Ian C. Scott, Zoe Paskins

**Affiliations:** 1 Haywood Academic Rheumatology Centre, Midlands Partnership NHS Foundation Trust, Stoke-On-Trent, UK; 2 School of Medicine, Keele University, Keele, UK; 3 School of Medicine, University of Exeter, Exeter, UK; 4 Academic Unit of Population and Lifespan Sciences, University of Nottingham, Nottingham, UK

**Keywords:** Anxiety, COVID-19, Depression, Gender, Mental health, Physical health

## Abstract

**Objective:**

To assess the longer term impact of the COVID-19 pandemic on the self-reported physical and mental health of people with inflammatory rheumatic diseases (IRDs).

**Methods:**

Two thousand twenty-four patients with IRDs were randomly selected from electronic health records. Survey invitations were sent (August 2021 coinciding with relaxation of UK COVID-19 restrictions) using SMS and postal approaches. Self-reported data included demographics, shielding status and physical (MSK-HQ) and mental health (PHQ8 and GAD7).

**Results:**

Six hundred thirty-nine people completed the survey (mean (SD) age 64.5 (13.1) years, 384 (60%) female). Moderate/severe impact of the pandemic on physical and mental health was reported by 250 (41%) and 241 (39%) respectively. One hundred seventy-two (29%) reported moderate/severe depression (PHQ8 ≥ 10) and 135 (22%) moderate/severe anxiety (GAD7 ≥ 10). Females reported greater impacts of the pandemic on physical health (44% vs 34%), mental health (44% vs 34%), arthritis symptoms (49% vs 36%) and lifestyle factors (weight gain and reduced exercise and physical activity) than males. The physical and mental impacts were less in people with RA compared with other IRDs. Physical health impacts did not differ between age groups, but younger patients reported greater impacts on mental health.

**Conclusion:**

The COVID-19 pandemic has had a significant impact on the physical and mental health of people with IRDs. These effects were greatest in females. Recovery needs to address the negative impact of the pandemic on lifestyle factors to minimise the long-term impacts for people with IRDs.

**Supplementary Information:**

The online version contains supplementary material available at 10.1007/s10067-023-06565-0.

**Table:** 

**Key Points** • *The pandemic had a significant impact on long term physical and mental health in almost 40% of people with IRDs.* • *The impact of the pandemic was greater in women for physical health, mental health and arthritis symptoms.* • *Many people reported negative pandemic impacts on lifestyle factors including weight and physical activity.*	

## Introduction

Concerns about the risk of COVID-19 infection led to guidelines advocating shielding for many people with inflammatory rheumatic diseases (IRDs) [1–3] together with a rapid shift to telemedicine and remote consulting [4]. Clinicians and patients had to balance the impact of ongoing disease activity with concerns around vulnerability to infection posed by escalating immunosuppressive treatment and glucocorticoids [5].

Particularly in the initial stages of the pandemic, studies in people with IRDs suggested higher levels of self-isolation and concordance with shielding advice than in the general population [6, 7], but also high levels of anxiety [6, 7]. Data from the cross-sectional REUMAVID study [8] which used an online survey across seven European countries observed that half of the patients assessed reported poor well-being, and 46.6% felt their health had changed for the worse during lockdown. This survey also found that, in common with the general population [9, 10], many people adopted unhealthy behaviours with reduced physical activity [8, 9] and increased smoking and alcohol consumption [8, 10], although other studies suggested these behaviours have varied over the course of the pandemic [11].

Whilst the direct health impacts of COVID-19 infection in terms of hospitalisation and death were higher in men than women, [12] data suggest that the indirect impacts of COVID-19 pandemic are greater for women than men, particularly in terms of employment loss and economic impacts [13]. Population data on the indirect effects of the COVID-19 pandemic on physical and mental health do not show consistent associations of gender with physical activity [14] or psychological impact [15] of the pandemic and further data are needed.

Our aim was to evaluate the longer term impacts of the COVID-19 pandemic on the physical and mental health of people with IRDs and to establish if these impacts differ between IRD types, males and females, and age groups. This knowledge is crucial to understanding if there are groups of people with IRDs that require additional support to overcome the long-term pandemic impacts.

## Materials and methods

We undertook a cross-sectional survey. Potential participants were identified from the rheumatology electronic health care record “DIAgnostic and MONitoring Database (DIAMOND)” at Midlands Partnership NHS Foundation Trust. This database contains clinical information about diagnoses, patient encounters and medications. All patients on DIAMOND who were under active follow-up (clinical contact within 2 years and not discharged since last review) and with a clinician diagnosis of one of the four commonest IRDs (rheumatoid arthritis (RA), axial spondyloarthropathy (AxSpA), psoriatic arthritis (PsA) or systemic lupus erythematosus (SLE)) were eligible for consideration for inclusion. Two thousand twenty-four patients were randomly selected from DIAMOND and invited to participate either by SMS text message (which included the option to complete the questionnaire via an online link, email, paper or by telephone with a researcher) or postal letter. A reminder SMS was sent at 1 week, and reminder letters sent at 2 and 4 weeks.

### Data collection

Invitations were sent in August 2021 (to coincide with the relaxation of England national COVID-19 restrictions). The survey collected data on age, gender, self-reported IRD diagnosis (characterised as RA, PsA, ankylosing spondylitis/AxSpA, SLE or other) and global impact of the pandemic on physical and mental health, arthritis symptoms and work (each scored with a 5-level response option, from “not at all” to “severely”). Impact on arthritis was assessed using the MSK-HQ [16], and in addition for each domain, people were asked whether this was different to pre-pandemic (5-level response option from “much worse” to “much better”). Mental health was assessed using PHQ-8, with a score of ≥ 10 suggesting current depression [17] and the GAD-7 with a score of ≥ 10 suggesting current anxiety [18]. Participants were asked to rate loneliness using the University of California, Los Angeles (UCLA) 3-item loneliness scale, with higher scores indicating greater loneliness [19]. People were also asked to rate the impact of the pandemic on lifestyle factors such as alcohol consumption, smoking, weight, physical activity and exercise.

### Statistical analysis

Statistical analysis was performed using Stata 17.0. The sample of responders was summarized using frequencies and percentages with means and standard deviations (SD) or medians and quartile values as appropriate. The impact of the pandemic on arthritis symptoms, mental and physical health was compared across IRDs, genders and age groups using analysis of variance, Kruskal–Wallis and chi-squared tests as appropriate.

### Ethical approval

Ethical approval was obtained from Surrey Borders REC (Ref 21/PR/0867), and all participants provided informed consent.

## Results

Six hundred and thirty-nine people (from 2,024 invited) completed the survey, of whom 287 (45%) completed it online. The majority (444, 70%) of participants reported having RA, with 100 (15.8%) reporting PsA, 21 (3.3%) AxSpA and 13 (2.1%) SLE. Fifty-seven (9.1%) reported having more than one IRD (including 3 with SLE), and so, these were combined with the small number reporting SLE only into an SLE/multiple category (Table 1). Seven people either left the diagnosis question blank or did not report one of the conditions of interest and so were excluded from further analyses. Mean (standard deviation (SD)) age was 64.4 (13.1) years, and 380 (64.7%) were female. Five hundred and eighty-one (98% of those completing the item) reported themselves to be of White British ethnicity. Three hundred forty-four (58%) people reported being advised to shield.

**Table 1:** Patient demographics and pandemic impacts on physical and mental health in males with IRDs compared with females

	Female *n* = 380 (65%)	Male *n* = 207 (35%)	Total (*n* = 632)	*p*-value
Age (years) (mean (SD))	63 (14)	67 (12)	64 (13)	< 0.001
Employment status (*n* (%))				
Employed	95 (25)	48 (23)	143 (24)	0.057
Retired	206 (54)	131 (63)	343 (58)	
Other	78 (21)	28 (14)	107 (18)	
Advised to shield (*n* (%))	202 (56)	111 (58)	344 (58)	0.675
Moderate/severe impact on physical health (*n* (%))	160 (44)	69 (34)	250 (41)	0.027
Moderate/severe impact on arthritis symptoms (*n* (%)	180 (49)	73 (36)	275 (45)	0.004
MSK-HQ^a^ (mean (SD))	29.3 (11)	31.9 (11)	30.2 (12)	0.008
Moderate/severe impact on mental health (*n* (%))	164 (44)	56 (28)	241 (39)	< 0.001
PHQ-8 depression score (median (IQR))	5.9 (2, 11)	4 (1, 8)	5 (2, 10)	0.001
PHQ-8 depression score ≥ 10 (*n* (%))	119 (32)	45 (22)	172 (29)	0.012
GAD-7 anxiety (median (IQR))	5 (0, 10)	2 (0, 7)	4 (0, 8)	< 0.001
GAD-7 anxiety score ≥ 10 (*n* (%))	99 (26)	32 (16)	135 (22)	0.003
UCLA loneliness^b^ (median (IQR))	4 (3, 6)	4 (3, 6)	4 (3, 6)	0.005
Moderate/severe impact on work (*n* (%))	75 (48)	33 (38)	118 (45)	0.172
Moderate/severe impact on finances (*n* (%))	48 (17)	25 (15)	81 (17)	0.516

^a^MSK HQ—higher score indicates worse function

^b^UCLA loneliness 3-item measure—higher score indicates worse loneliness

### Impact of the pandemic on physical health

Sixty-five (11%) people reported having had COVID-19 infection. There were no statistically significant differences in age, gender or arthritis subtype between those who did and did not report COVID infection. Overall, 250 (41%) reported a moderate or severe impact of the pandemic on general physical health; proportionally, more female subjects reported this than male subjects (44% versus 34%, *p* = 0.027) (Table 1), and this proportion was higher in people who reported a previous COVID infection (54% vs 38% *p* = 0.034). No difference was seen in the proportion of people reporting a moderate/severe impact of the pandemic on physical health across IRD types (*p* = 0.25) (Table 2) or age groups (*p* = 0.24) (Supplementary data).

**Table 2:** Patient demographics and pandemic impacts on physical and mental health in different inflammatory rheumatic diseases

	RA *n* = 444 (70.3%)	Axial SpA *n* = 21 (3.3%)	PsA *n* = 100 (15.8%)	SLE/Multiple^a^ *n* = 67 (10.6%)	*p*-value
Age (years) (mean (SD))	67 (12)	57 (11)	57 (11)	63 (16)	< 0.001
Female (*n* (%))	277 (67)	9 (50)	53 (58)	41 (66)	0.208
Employment status (*n* (%))					
Employed	18 (19)	8 (44)	45 (48)	12 (19)	< 0.001
Retired	269 (64)	5 (28)	32 (34)	37 (59)	
Other	72 (17)	5 (28)	16 (17)	14 (22)	
Advised to shield (*n* (%))	253 (60)	10 (53)	42 (45)	39 (61)	0.041
Moderate/severe impact on physical health (*n* (%))	164 (38)	10 (48)	44 (45)	32 (49)	0.246
Moderate/severe impact on arthritis symptoms (*n* (%))	176 (41)	12 (57)	54 (56)	33 (51)	0.030
MSK-HQ^b^ (mean (SD))	31 (11)	28 (4)	31 (12)	25 (10)	< 0.001
Moderate/severe impact on mental health (*n* (%))	151 (35)	8 (38)	48 (50)	34 (53)	0.005
PHQ-8 depression score (median (IQR))	4 (1, 9)	6 (4, 14)	7 (3,13)	6 (3,14)	< 0.001
PHQ-8 depression score ≥ 10 (*n* (%))	102 (24)	7 (35)	39 (41)	24 (38)	0.002
GAD-7 anxiety (median (IQR))	3 (0, 7)	4.5 (1, 12)	6 (1, 11)	6 (2, 13)	< 0.001
GAD-7 depression score ≥ 10 (*n* (%))	79 (19)	6 (30)	27 (28)	23 (35)	0.006
UCLA loneliness^c^ (median (IQR))	4 (3, 6)	5.5 (4, 6)	4 (3, 6)	5 (3, 6)	0.057
Moderate/severe impact on work (*n* (%))	60 (37)	9 (69)	35 (56)	14 (56)	0.009
Moderate/severe impact on finances (*n* (%))	48 (15)	4 (24)	18 (21)	11 (20)	0.473

Table 2 indicates *n* (%) unless otherwise stated

^a^Including SLE and people reporting more than one IRD

^b^MSK HQ—higher score indicates worse function

^c^UCLA loneliness 3-item measure—higher score indicates worse loneliness

Female subjects were also more likely to report moderate/severe pandemic impacts on arthritis symptoms (49% vs 36.%, *p* = 0.004). Proportionally, fewer patients with RA reported moderate/severe pandemic impacts on arthritis symptoms than other IRDs (41% vs 51 to 57%; *p* = 0.03), but there was no significant difference across age groups (supplementary data; *p* = 0.115). People reporting COVID infection were more likely to report moderate/severe pandemic impacts on arthritis symptoms (60% vs 41%, *p* = 0.005).

**Fig. 1:**
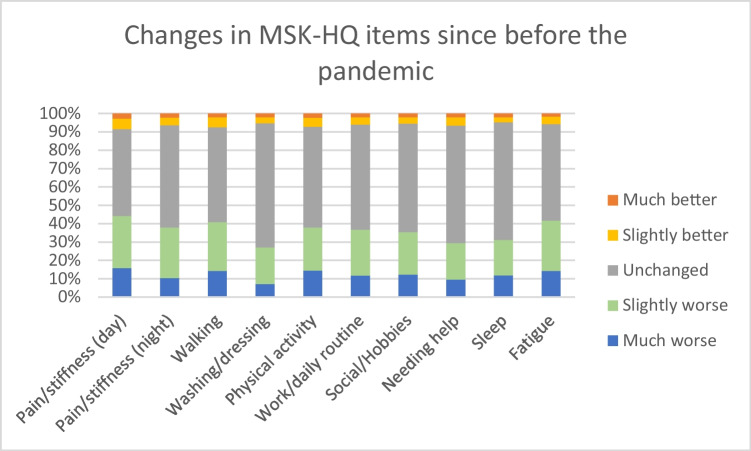
Changes in self-reported health since before the pandemic using MSK-HQ

Figure 1 highlights the differences across the domains of the MSK-HQ with people asked to rate changes in symptoms from before the pandemic, with up to 40% reporting worsening in daytime stiffness and fatigue. The impact of the pandemic on MSK-HQ items was more pronounced in those with AxSpA and multiple IRDs, particularly in relation to walking, social activities/hobbies and the need for help and sleep. The impact on night pain/stiffness and the need for help were also worse in females. There was little association with age, but the 40- to 49-year age group had slightly worse scores across all MSK-HQ items than other age groups (Supplementary data).

### Impact of the pandemic on mental health

Overall, 241 (39%) reported a moderate or severe impact of the pandemic on mental health. Moderate/severe pandemic impact on mental health was more common in female subjects than male subjects (44% of female subjects vs 28% of male subjects, *p* < 0.001) or of younger age (66% of < 40 year olds vs. 21% of ≥ 80 year olds, *p* < 0.001) and those reporting previous COVID infection (48% vs 36% *p* = 0.07), although this was not statistically significant in those reporting previous COVID infection. As for physical health, proportionally less patients with RA reported moderate/severe pandemic impacts on mental health than other IRDs (35% vs 38 to 53%, *p* = 0.005; Table 2). Overall, 172 (29%) reported moderate depression (PHQ8 ≥ 10) and 135 (22%) moderate anxiety (GAD-7 ≥ 10) which was lower in people with RA than other IRDs (*p* = 0.002 for moderate depression; *p* = 0.006 for moderate anxiety; Table 2), although there was not a significant difference for loneliness by IRD type. Female subjects were more likely to report current anxiety (*p* = 0.003), current depression (*p* = 0.012) or loneliness (*p* < 0.005) than male subjects (Table 1).

### Impact of the pandemic on work and finances

Proportionally, more younger people reported moderate/severe pandemic impacts on work (52% of < 40 year olds, 63% of 40–49 year olds, 16% of 70–79 year olds, *p* < 0.001). Proportionally, more younger people reported moderate/severe pandemic impacts on finances (25% of < 40 year olds, 32% of 40–49 year olds, 7% of ≥ 80 year olds, *p* < 0.001). No difference was seen in the proportion of male subjects vs female subjects reporting moderate/severe pandemic impacts on finances. People with RA were least likely and AxSpA most likely (37% vs 69%; *p* = 0.009) to report that the pandemic moderately/severely impacted their work. This may reflect the proportion of people with RA who reported they were retired, although we asked people to rate the pandemic’s impact on work/employment, and hence, work could reflect different activities.

### Impact of the pandemic on lifestyle factors

**Fig. 2:**
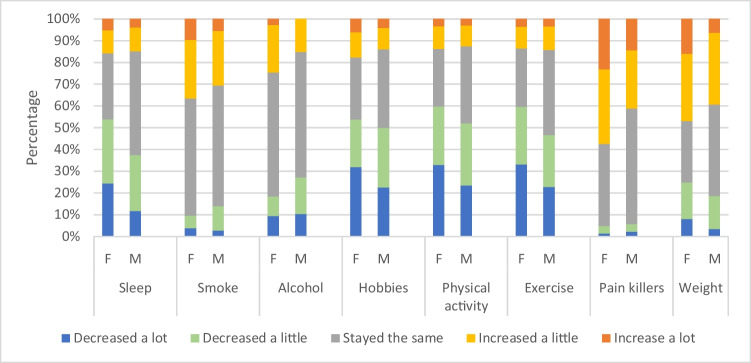
Impact of the pandemic on lifestyle factors in males and females with IRDs

Figure 2 illustrates the impact of the pandemic on lifestyle factors stratified by gender. Overall, females were more likely to report a decrease in “healthy” lifestyle factors such as physical activity and exercise and an increase in less “healthy” activities including painkiller use and smoking. Overall, more than 40% reported an increase in their weight, and this was more common in female subjects. The impact of the pandemic on lifestyle factors in different age groups and IRDs varied by lifestyle factor (supplementary data), but in general, younger people (particularly those aged 40–49 years) and females displayed increases in less “healthy” lifestyle characteristics compared to older people and males.

## Discussion

This survey, which represents one of the first analyses of the longer-term impacts of the COVID-19 pandemic, demonstrates a marked impact on the longer-term physical and mental health of people with IRDs, with 40% reporting that the pandemic had a moderate/severe impact on physical or mental health. Within our survey, the reported impacts of the pandemic on physical health were significantly greater in female subjects than male subjects and were significantly lower in people with RA compared to other IRDs. The impact of the pandemic on mental health was significantly greater in women and younger people. Our findings are broadly in line with studies examining the COVID19 pandemic’s initial impacts, such as the REUMAVID study which showed that 46.6% of respondents felt that their health had changed for the worse during lockdown [8], but our data shows that these negative impacts on health remain even 17 months after the pandemic’s onset. Our data on long-term mental health impacts also extends findings from a recent meta-analysis [15], which suggested that depression and anxiety showed consistently small but significant effects of lockdowns.

Understanding how the pandemic’s impacts on the health of people with IRDs differs by their age, gender and IRD type is crucial for planning future healthcare. Previous studies have highlighted the pandemic’s negative impacts on lifestyle factors such as physical activity, smoking and alcohol use [6, 8–11] in the general population as well as those with IRDs. Other studies suggest these lifestyle changes were greatest early in the pandemic, coinciding with lockdowns [9–11], but our work extends these findings, highlighting these changes persist in the long term. These negative impacts on lifestyle factors such as increased weight and reduced physical activity were greatest in female subjects, which if left unaddressed, it may lead to long-term health consequences including development of comorbidities such as cardiovascular disease (a common problem in people with IRDs). Our physical activity findings are in contrast to Christensen et al. [14] who undertook a meta-analysis of studies published in the first 6 months of the pandemic; this found a greater impact of increasing age on physical activity in the general population but that reductions in physical activity were similar for both genders.

To date, studies have reported heterogeneous findings regarding the impact of age and gender on COVID-19 pandemic outcomes [12–15]. Reasons for this are complex and likely to represent an interplay of biological, economic (such as work loss) and social factors (such as caring responsibilities). Nevertheless, there are policy imperatives [20] to try to ensure that the pandemic does not deepen pre-existing inequalities. An awareness of this gender impact can help in offering targeted advice (e.g. around exercise) and services that may help offset these negative lifestyle impacts.

Within our survey, the impact of the COVID-19 pandemic on physical and mental health appeared less in people with RA than other IRDs. There are a number of possible explanations for this. Amongst our respondents, people with RA were older, female and more likely to be retired, and therefore, the impact on work or other valued activities may have been reduced. Our recent longitudinal qualitative work examining the impact of lockdown on people with RA showed people that used previously acquired self-management techniques, including pacing and exercise to reduce the impact on wellbeing during the pandemic [21]. People with RA were also more likely to have been advised to shield earlier in the pandemic [3], which may have impacted on mental and physical health.

There are a several number of caveats that need to be considered when interpreting the results of this study. Firstly, this is a single-centre study, where the majority of participants reported themselves to be of white ethnicity, limiting the wider generalizability to other populations. Secondly, we did not have a control group and so are unable to directly compare our findings with a similar population without IRDs. Thirdly, we did not collect data on number or type of physician visits and so are unable to determine the impact of this on self-reported physical or mental health. Participation levels were moderate for a cross-sectional study, and it may be that there was differential response amongst people who were employed (although our mean age (67 years) was typical for that expected of an RA population). We attempted to broaden participation by using multiple methods of recruitment, including telephone and paper completion to support inclusion of people with limited health or digital literacy. Furthermore, the timing of this study (August 2021, almost 18 months into the pandemic and at the end of UK national restrictions) enables us to assess the longer-term pandemic impacts, rather than short-term effects of initial lockdowns, although there may be some risk of recall bias by asking people to reflect on how their health had changed since before the pandemic.

In summary, this study highlights that the COVID-19 pandemic had a broad impact on physical and mental health, even after almost 18 months of onset, and this effect was highest in females. Awareness of the differential impact of the pandemic is important to facilitate on targeting of health messages to those in greatest need to avoid longer term negative health impacts from the COVID-19 pandemic.

## Supplementary Information

Below is the link to the electronic supplementary material.

Supplementary file1 (DOCX 65 KB)

## Data Availability

The datasets used and/or analysed during the current study are available from the corresponding author on reasonable request.

## Declarations

### Disclaimer

The views expressed are those of the authors and not necessarily those of the NHS, the NIHR or the Department of Health and Social Care.
