# Supplementary material for: Exploring the longer-term impact of the COVID-19 pandemic on physical and mental health of people with inflammatory rheumatic diseases: a cross-sectional survey
Source: Clin Rheumatol. 2023 Mar 7;42(7):1903–9. doi: 10.1007/s10067-023-06565-0 (PMC9990972; doi:10.1007/s10067-023-06565-0)
Supplement: Supplementary file 1 — Supplementary file1 (DOCX 65 KB) [file 10067_2023_6565_MOESM1_ESM.docx]

Supplementary data

Lifestyle changes by IRD


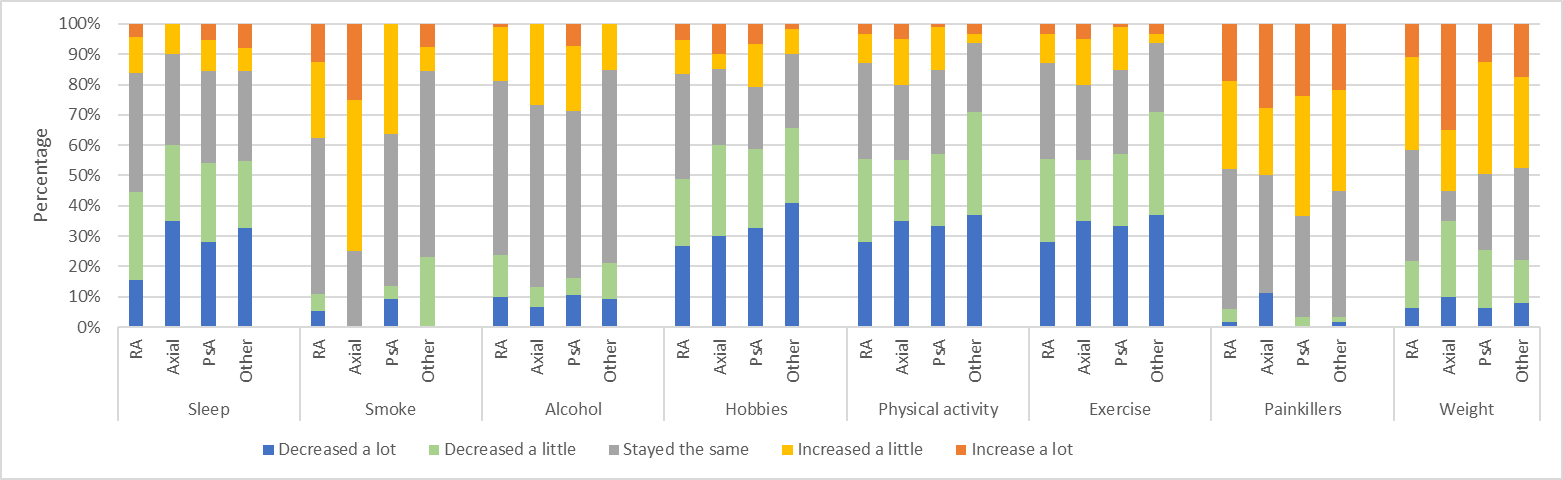


Lifestyle changes by age (years)

Impact on physical and mental health by age (years)

|  | <40  (n=29, 4.9%) | 40-49 (n=52, 8.8%) | 50-59  (n=112, 18.9%) | 60-69  (n=155, 26.2%) | 70-79  (n=178, 30.1%) | ≥80  (n=66, 11.2%) | p-value |
| --- | --- | --- | --- | --- | --- | --- | --- |
| Female | 21 (72) | 44 (85) | 77 (69) | 101 (66) | 97 (55) | 40 (62) | 0.003 |
| Employed  Retired  Other | 21 (72)  0 (0)  8 (28) | 31 (60)  0 (0)  21 (40) | 58 (52)  16 (14)  38 (34) | 28 (18)  93 (60)  34 (22) | 3 (2)  169 (95)  6 (3) | 2 (3)  63 (97)  0 (0) | <0.001 |
| Advised to shield | 13 (48) | 27 (52) | 56 (53) | 78 (54) | 106 (63) | 35 (58) | 0.45 |
| Moderate/severe impact on physical health | 11 (38) | 24 (47) | 53 (48) | 60 (40) | 63 (36) | 19 (32) | 0.28 |
| Moderate/severe impact on arthritis symptoms | 17 (59) | 28 (56) | 53 (47) | 65 (43) | 65 (38) | 27 (44) | 0.12 |
| MSK-HQ^b^ (mean (SD)) | 32(13) | 27 (12) | 30 (12) | 30.7 (12) | 31 (11) | 29.7 (11) | 0.198 |
| Moderate/severe impact on mental health | 19 (63) | 27 (62) | 51 (46) | 67 (45) | 45 (26) | 13 (21) | <0.001 |
| PHQ-8 depression score  (median (IQR)) | 8 (3,19) | 8 (4, 13) | 5 (2, 14) | 5 (2, 11) | 4 (2, 8) | 2.7 (0, 6) | <0.001 |
| PHQ-8 ≥ 10 | 12 (41) | 22 (42) | 39 (35) | 46 (30) | 36 (21) | 10 (15) | 0.001 |
| GAD-7 anxiety  (median (IQR)) | 6 (2, 15) | 6 (2, 12) | 4 (1, 13) | 5 (1, 10) | 2 (0, 7) | 1.1 (0, 5) | <0.001 |
| GAD ≥10 | 8 (28) | 14 (27) | 33 (30) | 42 (27) | 25 (14) | 9 (14) | 0.007 |
| 3 item UCLA Loneliness^c^  (median (IQR)) | 6 (4, 6) | 5 (3, 7) | 4 (3, 6) | 4 (3, 6) | 4 (3, 6) | 3 (3, 6) | 0.002 |
| Moderate/severe impact on work | 11 (52) | 26 (63) | 42 (57) | 23 (37) | 6 (17) | 0 (0) | <0.001 |
| Moderate/severe impact on finances | 7 (25) | 15 (32) | 26 (27) | 19 (17) | 4 (3) | 9 (7) | <0.001 |
